# The Metabolic Role of Mitochondria in the Perinatal Cardiac Development and Cardiovascular Diseases

**DOI:** 10.1002/EXP.20240414

**Published:** 2026-03-31

**Authors:** Minghao Li, Ruiyi Zhang, Xinyuan Li, Hefeng Huang

**Affiliations:** ^1^ Obstetrics and Gynecology Hospital Institute of Reproduction and Development Fudan University Shanghai China; ^2^ Research Units of Embryo Original Diseases Chinese Academy of Medical Sciences (No. 2019RU056) Shanghai China; ^3^ Medical Reproductive Center The Second Affiliated Hospital Soochow University Suzhou China; ^4^ Reproductive Medicine Center International Institutes of Medicine The Fourth Affiliated Hospital Zhejiang University School of Medicine Yiwu Zhejiang China; ^5^ Key Laboratory of Reproductive Genetics (Ministry of Education) Department of Reproductive Endocrinology Women's Hospital Zhejiang University School of Medicine Hangzhou China

**Keywords:** diabetic cardiomyopathy, heart development, heart failure, metabolism, mitochondria

## Abstract

The heart, as the central organ of the circulatory system, undergoes distinct metabolic transitions from embryonic development through postnatal maturation. A key aspect of this transition is mitochondrial maturation, which is influenced by changes in oxygen levels and metabolic substrates. These changes regulate cardiac structure and function by affecting signaling pathways and transcription factors. In cardiovascular diseases, mitochondria play a crucial role in altering metabolic patterns, contributing to pathological remodeling and worsening disease outcomes. This article explores mitochondrial roles in both cardiac development and disease progression, offering insights into potential therapeutic interventions.

## Graphical abstract

This review explores the critical role of mitochondria in heart development and cardiovascular disease. It highlights how mitochondrial maturation during embryonic‐to‐postnatal transition, regulated by oxygen and metabolic changes, shapes cardiac structure and function. The review also examines how dysfunctional mitochondria drive pathological metabolic shifts and remodeling in heart disease, offering insights for potential therapeutic interventions.

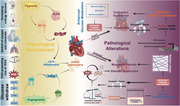

## 1 Introduction

Mounting epidemiological evidence from the American Heart Association reveals a staggering global burden of cardiovascular diseases (CVDs), accounting for approximately 19.41 million fatalities in 2021—representing a concerning 57% surge compared to 1990 baseline data. The current prevalence has reached an alarming 612.06 million cases worldwide, establishing CVDs as the preeminent public health challenge [1]. While traditional research on CVD risk factors has primarily focused on postnatal adverse exposures (such as obesity and smoking), recent studies have revealed that adverse metabolic exposures during early life stages are closely associated with the development of chronic metabolic diseases in adulthood [2]. Therefore, gaining a deeper understanding of metabolic changes during cardiac development and pathological processes holds substantial scientific and clinical significance. As a crucial organ for maintaining the supply of oxygen and nutrients throughout the body, the heart is the central hub of the circulatory system and begins to develop early in embryonic stages (starting at E7.5 in mice and at E21 in humans) [3, 4]. During perinatal development and throughout various pathophysiological conditions in cardiovascular diseases, the heart exhibits a unique metabolic switch distinct from other organs. Mitochondria are the structural basis for this shift. As a constantly contracting and pumping organ, the heart relies heavily on mitochondria, which account for approximately one‐third of the volume of cardiomyocytes, underscoring their pivotal role in cardiac function [5, 6]. But mitochondria were once merely regarded as the ‘engines’ for cardiac contraction. Actually, beyond their essential role in supplying adenosine triphosphate (ATP) for myocardial contraction, mitochondria are integral to energy metabolism as central organelles [7]. They are instrumental in regulating energy metabolism through their biogenesis, subcellular localization, maintenance of mitochondrial hemostasis, and dynamic behavior, which allows the heart to alternate its energy metabolism between anaerobic glycolysis and fatty acid oxidation (FAO) in response to different fuel supplies and oxygen conditions [8, 9].

For example, at the prenatal stage, the fetal heart with tubular immature mitochondria, which has relatively lower energy demands and relatively low‐oxygen supply, primarily uses anaerobic glycolysis, the most oxygen‐efficient process, to supply energy [10]. After birth, with the onset of pulmonary circulation and subsequently greater blood volume in the left heart, energy demands rise, accompanied by increased oxygen availability, making FAO, which produces more energy, the preferred energy source for the postnatal cardiomyocytes with mature mitochondria with abundant cristae [11]. However, in addition to the energy supply changes due to fuel switching, it's progressively found that the substrates themselves, such as glucose and fatty acids, as well as intermediate metabolites of related metabolic pathways (e.g., the pentose phosphate pathway (PPP), polyol pathway, and hexosamine pathway, etc.), are also relevant for cardiac development and survival of cardiomyocytes under stress conditions [12, 13]. Relevant metabolites can not only alter the activity of associated signaling pathways but also influence the activity of epigenetic enzymes or the availability of donors, thereby affecting the epigenome of cardiomyocytes and ultimately impacting gene expression [14, 15, 16]. This indicates that mitochondrial maturation critically orchestrates cardiac development by shifting energy metabolism from embryonic glycolysis to postnatal fatty acid oxidation (supplying ATP for contraction). This process drives structural remodeling (transition from cardiomyocyte hyperplasia to hypertrophy, myofibril assembly) and functional maturation through calcium signaling regulation (stabilizing excitation‐contraction coupling) and ROS homeostasis (via upregulated SOD2). Defective maturation triggers apoptosis, causing ventricular wall thinning and reduced cardiac output. Such pathology can originate from four interconnected insults: (1) inherited defects in mitochondrial biogenesis regulators or metabolic enzymes, (2) intrauterine stressors like chronic hypoxia or maternal hyperglycemia disrupting maturation signals, (3) postnatal deficiencies in fatty acids or essential micronutrients, and (4) teratogen exposure causing irreversible mitochondrial DNA damage. These insults converge into bioenergetic failure and oxidative stress, culminating in congenital heart defects or infantile cardiomyopathy.

In the progression of various heart diseases, mitochondria also exhibit similar metabolic regulatory roles. For example, during the onset of heart failure (HF), mitochondrial biogenesis is impaired, and fission increases, leading to changes such as reduced mitochondrial size and fragmentation [17, 18]. This transformation parallels the shift in cardiomyocyte metabolism from oxygen‐demanding FAO to more oxygen‐efficient glycolysis. While this change temporarily sustains the heart's energy supply, the accumulation of various metabolites may exacerbate myocardial remodeling and functional impairment [19, 20].

In this review, we elucidate the role of mitochondria in sustaining energy supply in cardiomyocytes by sensing cellular energy status throughout heart development and the progression of cardiovascular diseases. We examine their functions in the physiological and pathological transitions of metabolic substrates, while also exploring their potential as therapeutic targets for intervention in diverse cardiovascular conditions.

## 2 The Role of Mitochondria and Metabolic Shift in Cardiac Development

It is well‐established that during the perinatal development of the heart, myocardial energy fuels shift from glucose to fatty acids, and metabolic processes from anaerobic glycolysis to fatty acid oxidative phosphorylation [21]. However, this metabolic transition is neither absolute nor complete, primarily manifesting as a shift in the proportion of the heart's reliance between the two metabolic processes [22]. Prenatally, the heart employs glycolysis and oxidative phosphorylation in roughly equal proportions (about 50% each), whereas postnatally, approximately 80% of myocardial energy demands are met through fatty acid oxidation, with the remainder supplied by glycolysis. Previously, these mitochondrial‐driven transformations were viewed as definitive markers of myocardial maturation. However, emerging research indicates that mitochondria and metabolic transitions not only reflect developmental progression but also play a regulatory role in guiding substrate development [23, 24].

### 2.1 Triggers of Mitochondrial and Metabolic Shift

#### 2.1.1 Increasing Energy Demand and Substrate Switch

**FIGURE 1:**
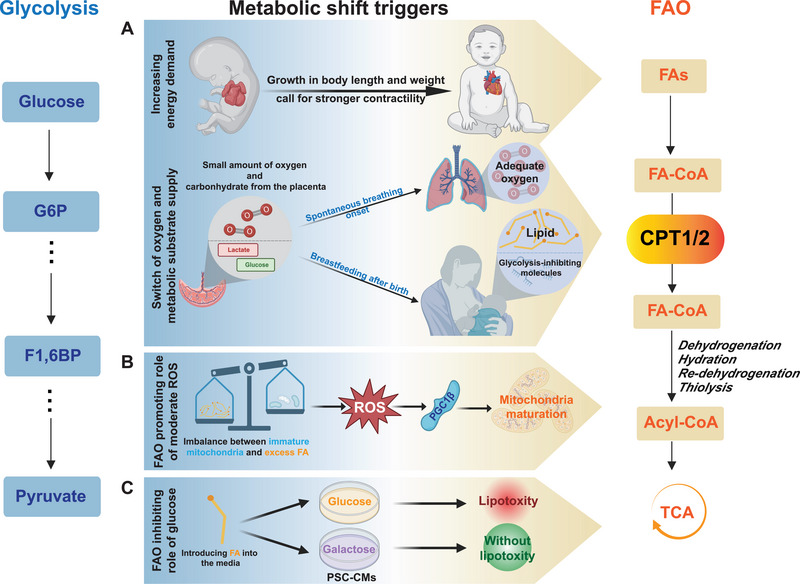
Triggers of metabolic shift during perinatal heart development. The increasing energy demands with physical growth, changes in oxygen and metabolic substrate supply, the controllable production of reactive oxygen species caused by “fuel‐engine” imbalance, and the gradual withdrawal of FAO‐inhibiting carbohydrates collectively drive the metabolic shift during the perinatal heart development. FAs, fatty acids; FA‐CoA, fatty acyl‐CoA; CPT1/2, carnitine palmitoyl transferase 1/2; acyl‐CoA, acetyl‐coenzyme A; G6P, glucose‐6‐phosphate; F1‐6BP, fructose‐1,6‐bisphosphate; ROS, reactive oxygen species; PGC1β, peroxisome proliferator‐activated receptor gamma coactivator 1β; PSC‐CMs, pluripotent stem cell‐derived cardiomyocytes. Created with BioRender.com, publication license VZ288F9CHY.

During perinatal development, various factors synergistically promote mitochondrial biogenesis and associated metabolic transitions. A key factor is the onset of independent breathing after birth, which opens the pulmonary circulation and markedly increases venous return to the left heart [25, 26]. Additionally, the neonatal period, characterized by rapid growth in body length and weight, calls for enhanced cardiac contractile function to meet these physiological needs (Figure 1A). While glycolysis prior to birth is oxygen efficient, it yields relatively modest energy output (two ATP molecules per glucose molecule). In contrast, although FAO demands considerably more oxygen, it generates substantially greater energy (120 ATP molecules per 16‐carbon fatty acid molecule). After birth, as newborns transition to independent respiration, blood oxygen levels do not constrain the ability to utilize fatty acids effectively of cardiomyocytes. Secondly, prior to birth, the fetus predominantly relies on metabolic substrates provided by maternal circulating lactate and glucose, the former of which is frequently converted to pyruvate within the cells and enters the tricarboxylic acid (TCA) cycle, whereas the latter primarily undergoes anaerobic glycolysis. Breastfeeding significantly induces carbohydrate deprivation in the infant circulation postnatally, while the fat content in breastmilk increases the levels of fatty acids and lipoproteins in the bloodstream, providing adequate fuel reserves for the establishing FAO [27]. In addition to transition in conventional metabolic fuels, some small molecules in breast milk, such as Let‐7 microRNA, can exert their effects by targeting and cleaving transcripts of key enzymes involved in glucose metabolism and modulating their translation [28, 29] (Figure 1). This mechanism not only drives the metabolic shift from glucose utilization to FAO but also supports the maturation of cardiomyocytes.

#### 2.1.2 FAO Promoting Role of Moderate Oxidative Stress

The transient spike in fat intake resulting from breastfeeding, juxtaposed with the relatively protracted process of mitochondrial biogenesis and maturation during the postnatal period—where a considerable quantity of TCA cycle intermediates is sustained through replenishment reactions—gives rise to an imbalance between “fuel” and “engine” [30]. This dissonance prompts stress responses within the mitochondria, culminating in the production of a limited, yet controllable, amount of reactive oxygen species (ROS) [31]. Unlike the excessive generation of detrimental ROS under pathological conditions, the relatively regulated ROS production induced by the imbalance between “fuel” and “engine” in this context not only does not lead to mitochondrial swelling and fragmentation, but also promotes mitochondrial biogenesis and the maturation of cardiomyocytes. The relatively regulated production of ROS in this context is advantageous for mitochondrial biogenesis and the maturation of cardiomyocytes [32, 33, 34]. ROS can enhance the expression of peroxisome proliferator‐activated receptors (PPARs) and peroxisome proliferator‐activated receptor γ coactivator (PGC) through pathways such as adenosine monophosphate activated protein kinase (AMPK), mitogen activated protein kinase (MAPK), and transcription factors like nuclear factor kappa B (NF‐κB), as well as via its own direct oxidative properties [35]. Both PPARs and PGC are instrumental in promoting mitochondrial biogenesis, facilitating mitochondrial fusion, and regulating the expression of key enzymes involved in FAO (Figure 1B). Considering the relatively hypoxic environment in which prenatal cardiomyocytes exist, the expression of PGC1α is upregulated through the synergistic regulation of ROS and hypoxia‐inducible factor 1‐alpha (HIF1α) [36]. This process enhances early mitochondrial biogenesis and fusion within these cardiomyocytes, effectively inhibiting the overactivation of the Calcineurin and Notch1 signaling pathways [37, 38]. As a result, it prevents abnormal differentiation of cardiomyocytes and malformations in vascular structures, which is critical for the proper differentiation of early cardiomyocytes. Under the regulation of PGC1α, components encoded by both nuclear DNA (nDNA) and mitochondrial DNA (mtDNA) assemble into a relatively complete mitochondrial structure, facilitating normal replication and transcription of mtDNA, as well as the expression of mitochondrial ribosomal proteins [39]. However, the expression of a series of essential functional molecules—such as key enzymes involved in FAO, oxidative phosphorylation coupling proteins, and components that maintain mitochondrial membrane potential—is upregulated by PGC1β, which is activated by controllable production of ROS postnatally [40]. In addition to the synergistic induction of PGC upregulation by ROS and HIF1α under hypoxic conditions, the first 24 h post‐birth are characterized by low blood glucose levels and an increase in the influx of carbohydrates that replenish TCA cycle intermediates. This state of energy deprivation elevates the AMP/ATP ratio, triggering AMPK activation, which subsequently drives the synchronized upregulation of PGC1α and PGC1β [41]. This coordinated expression is critical for the development of the cardiac conduction system; any deficiency in either factor can result in bradycardia, conduction disturbances, and ultimately HF [42].

#### 2.1.3 Deprivation of FAO‐Inhibiting Carbohydrates

Another compelling perspective posits that, beyond a myriad of intricate regulatory mechanisms, glucose itself may play a role in inhibiting cardiac maturation through a variety of pathways [43]. Thus, the postnatal deprivation of carbohydrates can significantly promote mitochondrial biogenesis, thereby advancing the functional and metabolic maturation of the heart [44]. In a study conducted on pluripotent stem cell‐derived cardiomyocytes (PSC‐CMs), Correia et al. demonstrated that treatment with glucose led to pronounced lipotoxicity when the substrate was shifted to fatty acids. In contrast, substituting glucose with galactose not only alleviated this lipotoxic response but also substantially promoted FAO [30] (Figure 1C). The intrauterine environment of the fetus, predominantly nourished by the placenta, provides a relative scarcity of fatty acids in the fetal circulation while ensuring an ample supply of carbohydrates. Thus, the inhibitory role of glucose on myocardial maturation serves to avert premature cardiac development, thereby mitigating the risk of insufficient fatty acid supply and subsequent energy deprivation. As a crucial bypass of glycolysis, what the PPP generates is essential for nucleotide synthesis. These metabolites serve as fundamental substrates for the replication of regenerative fetal cardiomyocytes, thereby fostering a hyperplastic immature myocardial phenotype while suppressing the hypercontractile mature phenotype [45]. Furthermore, the end product of anaerobic glycolysis—lactate—holds considerable significance in curbing myocardial maturation. In addition to supplying a rapid and efficient energy source for cardiomyocyte proliferation, lactate stabilizes hypoxia‐inducible factors (HIFs), particularly HIF1α, thereby enhancing lactate dehydrogenase A (LDHA) expression, which activates the mammalian target of rapamycin (mTOR) signaling pathway [46, 47]. This activation of the related pathway not only facilitates the progression of cardiomyocytes from the G1 to the S phase as well as promotes robust cellular proliferation. Additionally, it has been observed that elevated intracellular calcium concentrations, linked to contraction, can significantly enhance mitochondrial biogenesis [48].

### 2.2 Mitochondrial Maturation and Its Developmental Role

**FIGURE 2:**
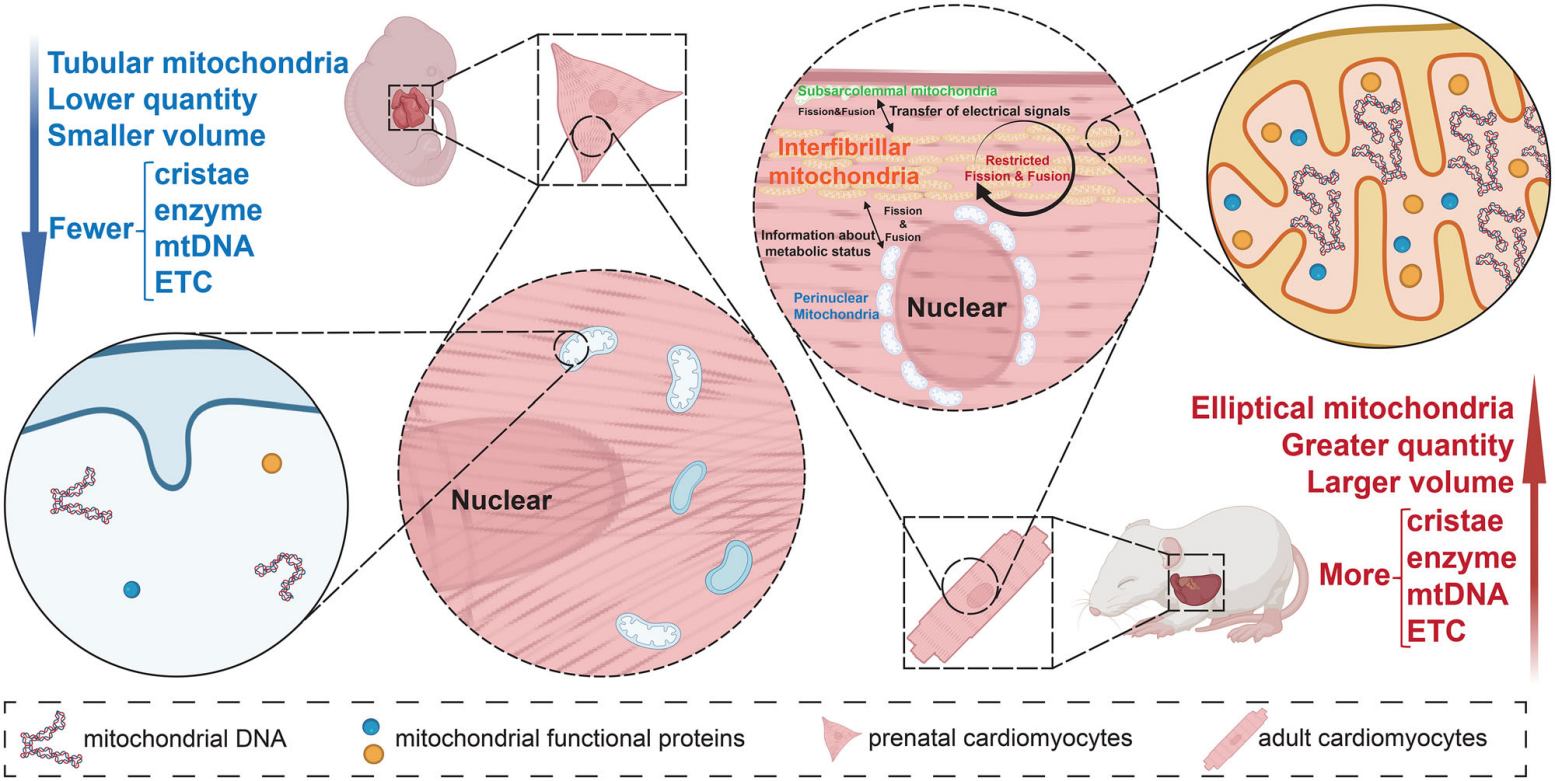
Changes in mitochondria pre‐ and post‐ myocardial maturation and metabolic shift. The functional structure and components of mitochondria, such as cristae, metabolic enzymes, mtDNA, and the electron transport chain, increase, and the distribution shifts from cytoplasmic mitochondria to three specialized groups: interfibrillar, subsarcolemmal, and perinuclear mitochondria. These subtypes can exchange signals through mitochondrial dynamics, with the interfibrillar group primarily providing energy for cardiac contraction, while its internal fission and fusion are restricted. mtDNA, mitochondrial DNA; ETC, electron transport chain. Created with BioRender.com, publication license ZT288F9B1E.

Driven by the above series of incentives, the mitochondria in perinatal cells, as the core organelles of aerobic oxidation, will undergo significant changes to adapt to the metabolic transition from anaerobic glycolysis to fatty acid aerobic oxidation. Mitochondrial changes can be encapsulated in several key aspects: firstly, the number of mitochondria increases significantly, alongside the complete development of the inner membrane (Figure 2). Throughout this process, mitochondrial biogenesis mediated by the PPAR and PGC families, as well as the fusion of early mitochondria, plays a critical role [49].

#### 2.2.1 Biogenesis and Homeostasis Maintenance of FAO‐Programmed Mitochondria

In the early to mid‐stages of cardiac development (at E10 in mice and E35 in humans), a relatively hypoxic environment activates HIF1α, which in turn promotes the transcription of KLF4. These two conserved transcription factors enhance the expression of PPAR and PGC (mainly PGC1α) family transcription factors. Among them, the latter serves as an anchor, tethering the former to the nuclear retinoic acid X receptor (RXR), thus forming a transcriptional regulatory complex [50]. The complex connected by PGC1α activates the expression of genes encoded on nDNA related to mitochondrial structure and function (such as fusion related genes: mitofusin (MFN), optic atrophy 1 (OPA1)), and promotes the transcription and translation of mtDNA, facilitating the formation of early mitochondrial structures and the expression of ribosomal proteins within them, thereby providing a structural foundation for the transition from early anaerobic glycolysis to aerobic oxidation [51]. However, as the heart structure progressively matures (at E13 in mice and during gestational weeks 7–8 in humans), the enhanced oxygen supply resulting from angiogenesis leads to a decrease in lactate levels, which in turn gradually diminishes the stability of HIF1α. Notably, this reduction in HIF1α activity does not hinder the differentiation of cardiomyocytes; rather, it plays a crucial role in their maturation [52]. Gentillon et al. demonstrated that the inhibition of HIF1α using FM19G11 effectively promotes the maturation of PSC‐CMs.

**FIGURE 3:**
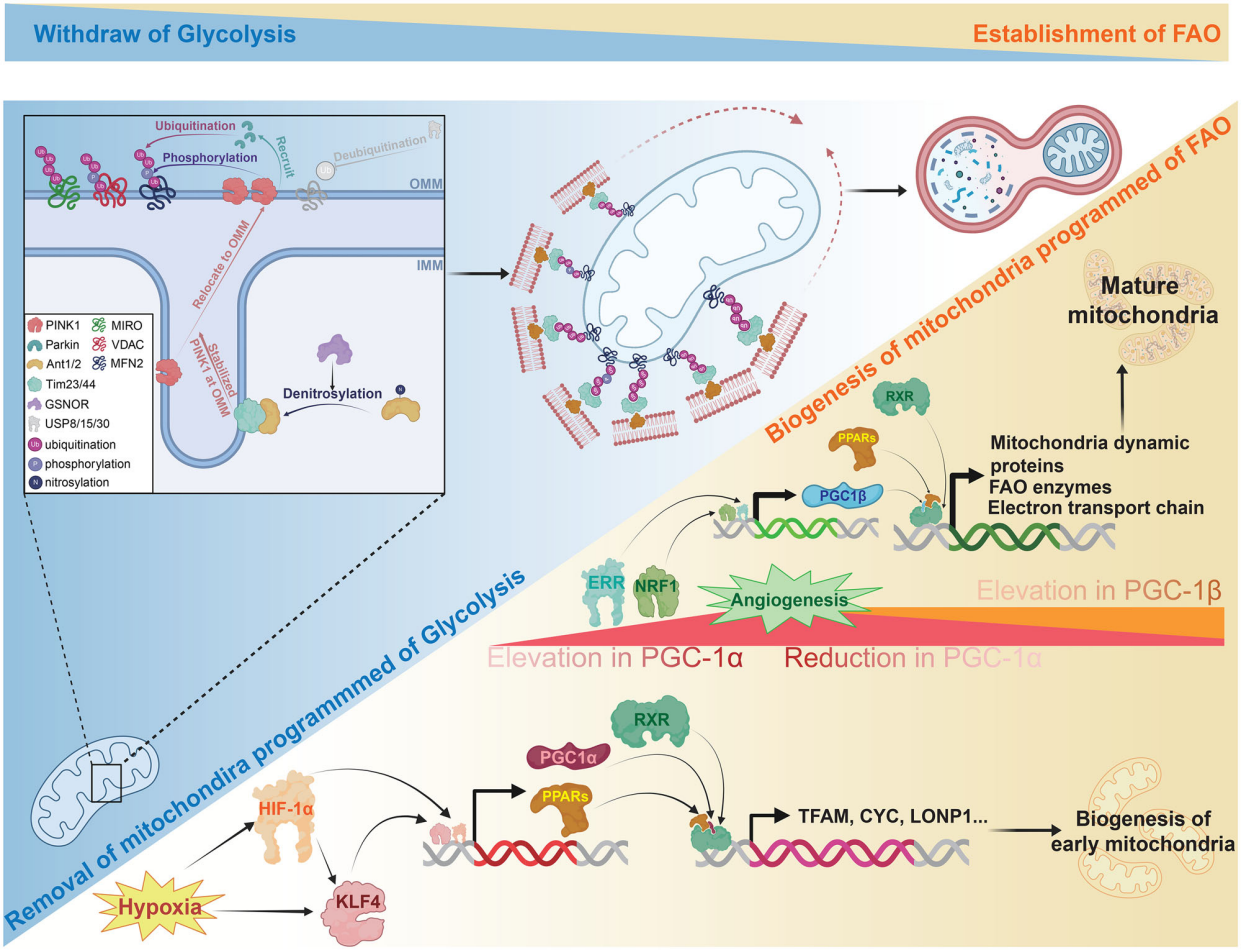
The essence of cardiac metabolic transition is the maturation of mitochondria. During metabolic maturation, mitochondria programmed for glycolysis will be gradually eliminated through ubiquitin‐mediated mitophagy, while mitochondria programmed for fatty acid oxidation will gradually emerge and mature under the regulation of a series of transcription factors. HIF‐1α, hypoxia‐inducible factor 1 alpha; KLF4, Krüppel‐like factor 4; PGC1α, peroxisome proliferator‐activated receptor gamma coactivator 1 alpha; RXR, retinoid X receptor; PPARs, peroxisome proliferator‐activated receptors; TFAM, mitochondrial transcription factor A; CYC, cytochrome c; LONP1, LON peptidase 1; ERR, estrogen‐related receptor; NRF1, nuclear respiratory factor 1; FAO, fatty acid oxidation. Created with BioRender.com, publication license HE288F99XC.

The expression of PGC1β mainly depends on transcription factors such as estrogen‐related receptor (ERR) and nuclear factor erythroid 2‐related factor (NRF) instead of HIF1α [53]. Therefore, the downregulation of HIF1α does not affect the simultaneous upregulation of PGC1β, which regulates the gene expression related to further mitochondrial maturation by anchoring PPAR and RXR to form transcriptional regulatory complexes. This facilitates the transcription of genes associated with fatty acid oxidative metabolism and mitochondrial dynamics, thereby ensuring the maintenance of mitochondrial homeostasis and the enhancement of FAO capacity. In addition to increasing the proportion of mitochondria involved in FAO, the withdrawal of HIF1α also prevents the excessive activation of PGC1α, thereby averting the abnormal upregulation of proteins such as MFN2 and OPA1, which mediate excessive mitochondrial fusion [54] (Figure 3). As previously noted, the pronounced upregulation of replenishment responses after birth may induce carbohydrate deprivation. Studies indicate that during this “energy stress,” the upregulation of dynamin‐related protein (DRP1) expression facilitates a more effective balance between mitochondrial fission and fusion dynamics [55]. In contrast, excessive and aberrant fusion could worsen the detrimental effects of carbohydrate deprivation on cardiomyocytes. Thus, the attenuation of HIF1α is of paramount importance for the metabolic transition of cardiomyocytes during the postnatal period.

Secondly, the mitochondrial contents—including mtDNA, the electron transport chain (ETC), and critical enzymes involved in fuel oxidation—will undergo significant augmentation. This enhancement is contingent upon the maintenance of mitochondrial homeostasis, which is facilitated by various factors, including mitochondrial dynamic related proteins such as MFN2, kinase like PTEN Induced Kinase 1 (PINK1), and proteases such as LON peptidase 1 (LONP1) [56, 57]. Each of these components may be upregulated and contributes uniquely to the preservation of mitochondrial stability. Among these, the first two primarily preserve mitochondrial homeostasis through the regulation of mitochondrial dynamics. As for LONP1, an enzyme responsible for the degradation of misfolded or damaged proteins within the mitochondria, it is pivotal in sustaining mitochondrial homeostasis throughout the developmental process. When LONP1 malfunctions, the resulting accumulation of misfolded proteins perturbs mitochondrial homeostasis, which in turn activates stress response pathways such as eIF2α. The downstream effector activating transcription factor 4 translocates to the nucleus, where it curbs the expression of mitochondrial transcription factor A (TFAM) and a host of nuclear DNA‐encoded mitochondrial oxidative‐related proteins. Consequently, downregulated TFAM inhibits the proper expression of a range of mitochondrial functional proteins encoded by mtDNA, which further exacerbates the accumulation of misfolded proteins, thereby creating a vicious cycle [58]. This also underscores the significance of LONP1 not only in maintaining mitochondrial homeostasis but also in ensuring the maturation of mitochondrial function and metabolism in cardiomyocytes [59]. The augmentation of contents within mitochondria will precipitate a series of notable structural alterations, including an increase in mitochondrial volume, a transition from a tubular to an elliptical morphology, and most critically, a marked enhancement in the number of mitochondrial cristae. The expansion of the inner membrane surface area, coupled with the distinctive architecture of the cristae, facilitates the accommodation of a greater number of respiratory chain complexes and ATP synthase, ensuring that key enzymes and proteins of the ETC are more concentrated, significantly enhancing the efficiency of electron transfer. Furthermore, this structural reorganization aids in establishing a robust proton gradient across the membrane, thereby optimizing the functionality of ATP synthase [60].

#### 2.2.2 Mitochondrial Dynamic Regulation and Removal of Glycolysis‐Programmed Mitochondria

In the realm of mitochondrial dynamics, fusion‐related proteins play a pivotal role in mediating the interaction between distinct mitochondria. MFN1 and MFN2 are primarily responsible for facilitating outer membrane fusion, whereas OPA1 orchestrates the fusion of the inner membrane. This process not only promotes the integration of essential functional proteins across different mitochondria but also enhances the overall volume and surface area of the inner membrane [61]. Consequently, this synergistic action enables cells to optimize their energy production capabilities. The absence of MFN1/2 expression often leads to the shrinkage and fragmentation of mitochondria, subsequently causing energy supply disruptions in cells. Furthermore, in increasingly mature cardiomyocytes, although the dense muscle fibers restrict the space available for mitochondrial fusion and fission, a small portion of mitochondria can still move between different groups and fuse with them, facilitating communication and information transfer among the various mitochondrial populations [62]. Fission‐related proteins predominantly encompass DRP1 and a variety of mitochondrial membrane receptors, including mitochondrial fission 1 protein (Fis1), mitochondrial fission factor (MFF), and mitochondrial dynamics protein MID49/51 (MiD49/51). Upon recruitment to specific receptor sites, DRP1 harnesses energy from GTP hydrolysis to adopt a constrained circular conformation, facilitating the constriction and eventual strangulation of mitochondria [63]. This fission mechanism is crucial not only for the removal of dysfunctional mitochondria and the clearance of metabolic byproducts but also plays a significant role in promoting the biogenesis of new mitochondria [64, 65]. As previously noted, the enhancement of mitochondrial fission can facilitate cardiomyocytes in enduring the transient “carbohydrate deprivation” experienced shortly after birth. However, excessive fission under normal physiological conditions may disrupt energy supply, potentially resulting in oxidative stress and subsequent apoptosis [66]. Conversely, insufficient fission can lead to the accumulation of mitochondrial metabolic waste and a heightened vulnerability to damage, thereby undermining cellular integrity and function.

In addition to their inherent function in mitochondrial integration and homeostasis maintenance, MFN2 and DRP1 are equally vital in mediating the clearance of damaged or dysfunctional mitochondria during cardiac development and the progression of cardiovascular diseases [67, 68]. The removal mediated by mitochondrial outer membrane proteins, represented by MFN2 primarily facilitates mitochondrial turnover throughout the cell [69]. This turnover is characterized not by changes within the same group of mitochondria, but rather by the clearance of old mitochondria and the generation of new ones, which means that the essence of metabolic shift is mitochondrial transformation [25] (Figure 3). In contrast, DRP1 typically eliminates damaged or dysfunctional mitochondrial portions through a budding mechanism. Involvement of MFN2 in mitochondrial clearance is independent of its inherent role of mitochondrial fusion, fundamentally relying on its modification by PINK1. Dorn et al. reveal that point mutations at critical phosphorylated amino acid residues can lead to significant impairments in the clearance of fetal mitochondria, which consequently curb the biogenesis of mitochondrial programmed to FAO induced by PGC1β. Consequently, the introduction of this mutation during the perinatal period would result in significant disruptions in the maturation of mitochondrial and metabolic profiles in cardiomyocytes, which may lead to lethal damage such as progressive dilated cardiomyopathy and systolic dysfunction [70]. This dysfunction is primarily characterized by a decrease in mitochondrial abundance and number of cristae, a downregulation of oxidative phosphorylation‐related protein expression, withdrawal from glycolytic pathways, and impairments in the emerging of FAO [71]. However, studies have demonstrated that after the maturation of cardiac function and metabolic development, mutations that were lethal during the embryonic stage may subsequently lose their lethality and pathogenic potential. This discovery underscores the pivotal role of PINK1 mediated phosphorylation in regulating MFN2's function in mitochondrial quality control, while also emphasizing the essential significance of MFN2 mediated mitochondrial elimination during the metabolic transitions that occur in perinatal cardiac development.

The mitochondrial replacement that induces perinatal metabolic pattern shifts is orchestrated by proteins such as MFN2. In this context, the kinase PINK1, which is typically degraded by presenilin associated rhomboid‐like protein (PARL) at the inner membrane under physiological conditions, undergoes translocation to the outer membrane. Denitrosylation of adenine nucleotide translocator 1 and 2 (ANT1 and 2) by S‐nitrosoglutathione reductase (GSNOR) facilitates their association with translocase of the inner membrane 23 and 44 (Tim23 and 44) to form a stable complex [72]. This assembly enhances the accumulation of PINK1 on the outer mitochondrial membrane and subsequently recruits E3 ubiquitin ligases, such as Parkin, thereby promoting the autophagic sequestration of damaged mitochondria. This process is intricately regulated by multiple factors. Research indicates that in HeLa cells, the absence of Parkin leads to a compensatory upregulation of optineurin (OPTN), nuclear dot protein 52 kDa (NDP52), and Tax1‐binding protein 1 (TAXBP1), which collectively contribute to the ubiquitination of mitochondrial outer membrane proteins [73]. Furthermore, the ubiquitination capability of the first two proteins can be enhanced through phosphorylation or oligomerization during oxidative stress, thereby promoting the programmed elimination of mitochondria [74, 75]. This mechanism may partially explain how the postnatal controllable amount of ROS activates mitochondrial turnover and cardiomyocytes maturation. PINK1, localized on the outer mitochondrial membrane, phosphorylates several outer membrane proteins, including MFN2. Early postnatal activation of AMPK leads to phosphorylation of PINK1 at the Ser495 site, which significantly enhances its kinase activity and regulatory capacity over mitochondrial quality control during developmental metabolic transitions [76]. The recruitment and activation of Parkin facilitates the ubiquitination of various mitochondrial outer membrane proteins, including MFN2, voltage‐dependent anion channel (VDAC), and mitochondrial Rho‐GTPase [77, 78]. These ubiquitin modifications can then engage with adaptors possessing both ubiquitin‐binding and LC3‐interacting domains (LIR), such as neighbor of BRCA1 gene 1, nuclear dot protein 52, and Optineurin, thereby promoting their association with phagophore membranes that are decorated with LC3 [79, 80]. In this process, only multiple ubiquitination or the combination of ubiquitination and phosphorylation signals are recognized as stable signals by the adaptors, while single ubiquitin modifications are not recognized and degraded by ubiquitin‐specific proteases (such as USP8, USP15, and USP30) [81]. This ensures the precision of mitophagy and prevents random Parkin spread in the cytoplasm from triggering the removal of healthy mitochondria. Then, the phagophore will undergo fusion with lysosomes, under the guidance HDAC6, facilitating the gradual degradation of embryonic glycolytic mitochondria [82]. Simultaneously, an increase in both the quantity and proportion of FAO‐associated mitochondria, governed by PGC1β, enables cardiomyocytes to effectively accomplish their metabolic transition during the perinatal phase (Figure 3).

#### 2.2.3 Alterations in the Intracellular Distribution of Mitochondria

Apart from the changes in the mitochondria themselves, the distribution of mitochondria undergoes significant alterations during the maturation of cardiomyocytes, coinciding with the generation of myofilaments [83] (Figure 2). In immature prenatal cardiomyocytes, irregularly shaped tubular mitochondria are often dispersed haphazardly throughout the cytoplasm. As mitochondrial dynamics involve both fission and fusion, damaged or non‐functional mitochondrial components are continuously removed, while fusion processes gradually enhance metabolic activity. As cardiomyocytes undergo maturation, mitochondria exhibit a specialized distribution into distinct subgroups located at various sites, each assigned specific functions. Among these, interfibrillar mitochondria play a pivotal role in myocardial contraction. They typically form well‐organized network structures, intricately enveloped around the myofibrils situated between the Z‐lines. The dense arrangement of these myofibrils imposes significant constraints, resulting in a limited occurrence of fission and fusion among these mitochondria [84]. This configuration not only ensures functional integrity—where an expanded mitochondrial membrane surface area provides a sufficient reservoir of mitochondrial functional proteins—but also significantly enhances energy transfer efficiency, thereby satisfying the elevated energy demands of cardiomyocytes. In addition to supplying energy for the contracting myofibrils, the mitochondria distributed along the myofibrils also provide the energy necessary for calcium recovery following contraction, thereby ensuring the continuity of cardiac contraction [85]. In the coordination of contraction among different cardiomyocytes, subsarcolemmal mitochondria play a crucial role. They generate ATP to power ion pumps and channels on the cell membrane, influencing the propagation of action potentials [86]. Moderate levels of ROS production help maintain the electrophysiological properties of adult cardiomyocytes by regulating key channel proteins, intercellular connexins, and calcium‐handling proteins. For instance, ROS can enhance the activity of the sodium channel (NaV1.5) to accelerate the depolarization of the action potential, activate the PKC pathway to increase calcium influx mediated by L‐type calcium channels, and prolong the plateau phase by inhibiting the transient outward potassium current, thereby promoting calcium‐triggered contraction [87]. Additionally, ROS modulates the phosphorylation of Connexin 43 via the MAPK pathway to preserve gap junction coupling and enhances the calcium sensitivity of RyR2 through oxidation to improve contractile synchrony [88, 89]. These regulatory mechanisms ensure synchronized myocardial contraction, optimizing the direction and force of cardiac pumping for efficient blood ejection [90]. In contrast, excessive oxidative stress renders cardiac mitochondria highly sensitive to minor perturbations, a phenomenon termed mitochondrial criticality. This not only disrupts the coordinated contraction of cardiomyocytes but also promotes arrhythmogenesis by facilitating the propagation of localized depolarization [91].

The third type of mitochondria—perinuclear mitochondria—plays a pivotal role in myocardial remodeling across various pathological conditions. These mitochondria can sense the stress status of the myocardium and adapt their energy metabolism accordingly. By releasing an array of distinct metabolites, they influence the epigenetic modifications and transcriptional expression of key genes, ultimately driving both the physiological and pathological process of myocardial remodeling [92]. Zeng et al. demonstrated that postnatal upregulation of α‐ketoglutarate dehydrogenase (OGDH) in cardiomyocytes reduces α‐ketoglutarate (α‐KG) levels, leading to decreased activity of lysine demethylase 6B (KDM6B). This suppression impairs KDM6B's ability to erase H3K4me3 (linked to maturation‐related genes) and H3K27me3 (associated with cardiomyocyte proliferation) [93]. Consequently, proliferation‐related genes are gradually silenced, while maturation‐related genes are activated, driving the transition from a hyperplastic mode to a contractile mode. The intrauterine developmental stage is crucial for fetal myocardial metabolic transition, where maternal metabolic abnormalities can cause long‐term cardiac dysfunction and increased mortality from cardiovascular diseases in offspring [94, 95]. Notably, mitochondrial dynamics enable cardiomyocytes to maintain self‐renewal capacity via autophagy‐biogenesis homeostasis. This suggests that abnormal metabolites in pathological conditions may similarly influence long‐term cardiovascular health through genetic‐related mechanisms. For instance, offspring of patients with polycystic ovary syndrome (PCOS) are often exposed to elevated androgen levels during gametogenesis and embryonic development. Sinha et al. discovered that this abnormal hormonal exposure could lead to increased trimethylation of histone H3 at lysine 9 (H3K9me3), an epigenetic modification, on cardiovascular metabolic genes that should normally be upregulated. This, in turn, disrupts the proper expression of these genes, adversely affecting the normal metabolic function of the offspring's cardiovascular system [96].

In the alteration of mitochondrial distribution, distinct subtypes of mitochondria are often spaced apart due to the constraints of muscle fibers. This distance inevitably form an obstacle for communication between mitochondria [97]. However, they can still merge and communicate through the fusion of vesicles from different subtypes, facilitating the transfer of electrical signals, as well as the information about metabolic status. Importantly, this mechanism also offers a key advantage: it prevents the mixing of components from different mitochondrial populations, which could lead to dysfunction and decreased energy production efficiency, thereby ensuring the specialized roles of mitochondria [98].

Collectively, during perinatal cardiac development, mitochondria undergo three key adaptations to drive the metabolic shift from glycolysis to fatty acid oxidation (FAO). Increased energy demands, substrate availability changes, moderate oxidative stress, and carbohydrate deprivation trigger these changes. First, PPAR/PGC transcriptional complexes promote mitochondrial biogenesis and augment components like ETC complexes and FAO enzymes, enhancing oxidative capacity through structural changes like increased cristae. Second, balanced fusion (MFN1/2, OPA1) and fission (DRP1) integrate contents and remove damage, while PINK1/Parkin‐mediated mitophagy specifically clears glycolysis‐programmed fetal mitochondria. Third, mitochondria reorganize into specialized subpopulations (interfibrillar, subsarcolemmal, perinuclear) to optimize energy delivery for contraction, electrophysiology, and gene regulation, supporting functional cardiac maturation.

## 3 The Role of Mitochondria and Metabolic Shift in Cardiovascular Diseases

As an organ that continuously performs autonomous rhythmic contractions and has high energy demands with metabolic flexibility, the myocardium undergoes a series of metabolic changes in response to different diseases, particularly in situations of “fuel deficiency” or “fuel excess” [99]. As the central hub of energy metabolism, mitochondrial adaptation drives metabolic shifts. While pathological metabolic changes resemble perinatal transitions and involve partial fetal gene reactivation, they do not fully replicate fetal reprogramming due to incomplete structural synchronization. Structural differences in mitochondria compared to fetal cardiomyocytes further limit the functional impact of fetal gene expression. Short‐term, this alteration preserves cardiac ejection and shortening fractions, but long‐term, it promotes disease progression and ventricular remodeling through molecular mechanisms involving both adaptive and maladaptive pathways [100, 101].

### 3.1 Heart Failure

As a severe and terminal stage of various cardiovascular diseases, HF exhibits two important characteristics in energy metabolism: a reduction in energy production and a loss of metabolic substrate flexibility [102]. These two aspects are closely interconnected and mutually influential. The former primarily manifests as a significant decrease in ATP, and the ratio of phosphocreatine to ATP (approximately 40% reduction) in cardiomyocytes [103], which is not solely attributable to structural and functional alterations in mitochondria themselves. Crucially, creatine kinase (CK)—a key enzyme responsible for energy metabolism and a critical regulator of the phosphocreatine‐ATP energy buffering system—exhibits significantly reduced activity during heart failure, accompanied by a marked decline in the phosphocreatine/ATP ratio. This impairment leads to substantial deficits in energy storage and buffering capacity near myofibrils. Numerous studies have demonstrated that overexpression of myofibrillar CK (CKmyofib) and mitochondrial CK (CKmito) significantly improves left ventricular function and attenuates pathological remodeling through enhanced energy reserve capacity and amelioration of oxidative stress [104, 105].

**FIGURE 4:**
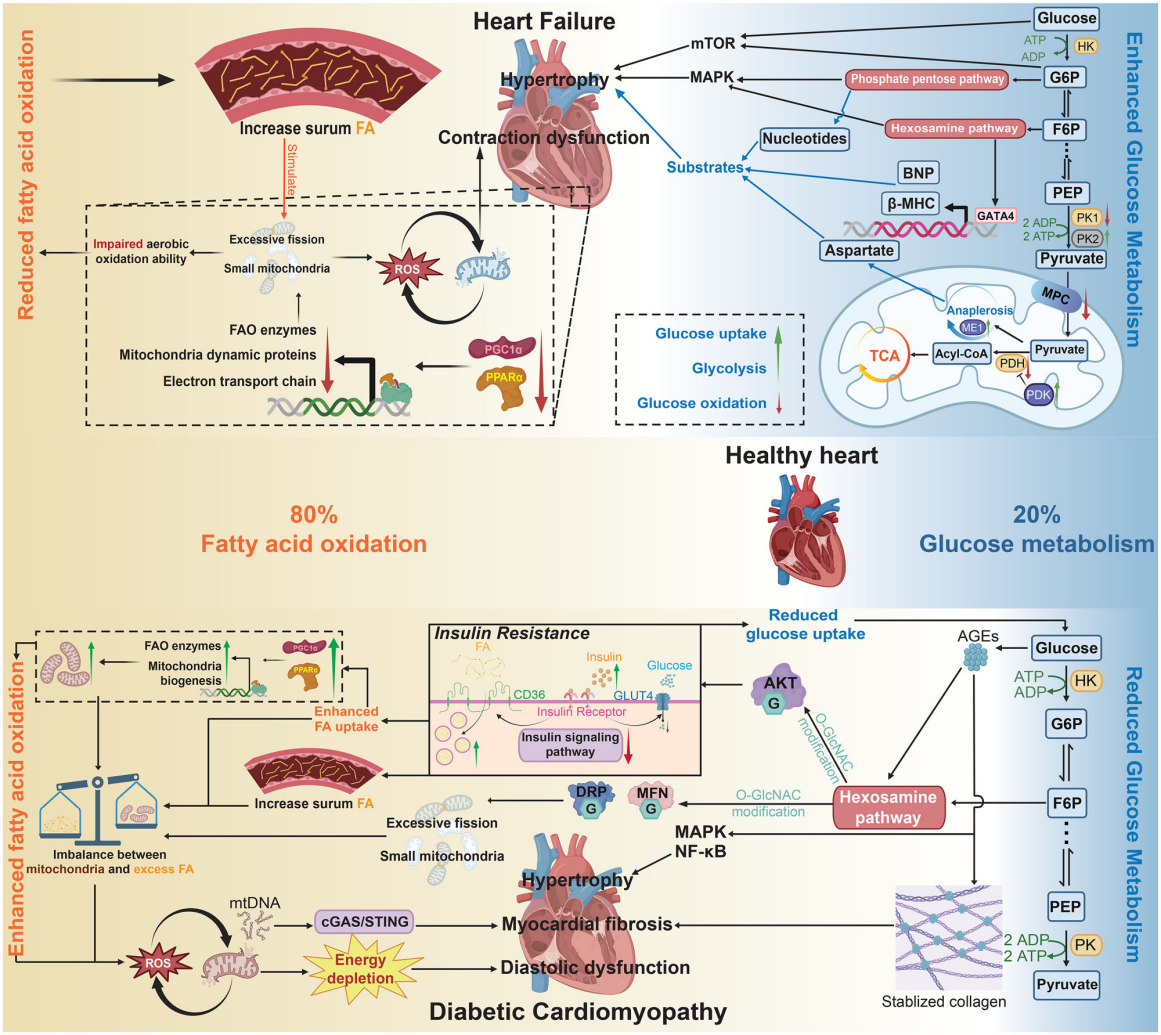
Heart failure and diabetic cardiomyopathy exhibit altered metabolic patterns compared to healthy hearts, along with changes in mitochondria and metabolic pathways. In heart failure, the upregulation of glucose uptake and glycolysis, coupled with a decrease in glucose oxidative metabolism, leads to cardiac hypertrophy through a series of metabolites. Meanwhile, the disruption of mitochondrial structure and insufficient fatty acid oxidation (FAO) result in contractile dysfunction. In diabetic cardiomyopathy, insulin resistance causes a reduction in glucose metabolism and an increase in fatty acid oxidation. The intermediate metabolites of glucose can lead to cardiomyocyte hypertrophy and fibrosis through complex mechanisms, while excessive fatty acid oxidation results in severe mitochondrial damage, contributing to significant myocardial fibrosis and diastolic dysfunction. PGC1α, peroxisome proliferator‐activated receptor gamma coactivator 1 alpha; PPARα, peroxisome proliferator‐activated receptor alpha; G6P, glucose‐6‐phosphate; F6P, fructose‐6‐phosphate; PEP, phosphoenolpyruvate; MPC, mitochondrial pyruvate carrier; PDK, pyruvate dehydrogenase kinase; PDH, pyruvate dehydrogenase; PK, pyruvate kinase; GATA4, GATA binding protein 4; BNP, B‐type natriuretic peptide; β‐MHC, beta‐myosin heavy chain; MAPK, mitogen‐activated protein kinase; mTOR, mechanistic target of rapamycin; AKT, protein kinase B; CD36, cluster of differentiation 36; MFN, mitofusin; DRP, dynamin‐related protein; cGAS, cyclic GMP‐AMP synthase; STING, stimulator of interferon genes; AGEs, advanced glycation end products. Created with BioRender.com, publication license EI288F9FOE.

The latter is reflected in the altered composition and proportions of energy metabolism substrates in cardiomyocytes compared to those in healthy ones. In normal cardiomyocyte energy metabolism, mitochondrial FAO accounts for approximately 80% of the energy production, while other substrate‐dependent forms, such as glycolysis, contribute only about 20%. Therefore, abnormalities in the structure and function of mitochondria, which are crucial for aerobic oxidation, become a primary cause of energy supply dysfunction in the progression of HF [106] (Figure 4).

One of the primary factors leading to dysfunction is the increased fission induced by changes in the expression patterns and activity of mitochondrial dynamics related proteins. This results in a reduction and fragmentation of mitochondria in cardiomyocytes, diminishing the inner mitochondrial membrane (IMM) area and providing a structural basis for the decreased capacity for aerobic oxidation [107]. In addition to the mitochondria in the heart, studies have also detected mitochondrial fragmentation in corresponding organs of patients with HF who experience reduced exercise capacity and renal failure [108, 109]. This suggests that mitochondrial defects play a critical role in the systemic pathophysiology of HF and may also be an important cause of damage to other systems affected by HF. In diseases such as HF and myocardial infarction, where “fuel insufficiency” is a key issue, the increase in mitochondrial fission is often seen as a mechanism to adapt to changes in energy status by effectively reducing the “engine displacement.” This adaptation aims to minimize oxidative stress response as much as possible [110].

**FIGURE 5:**
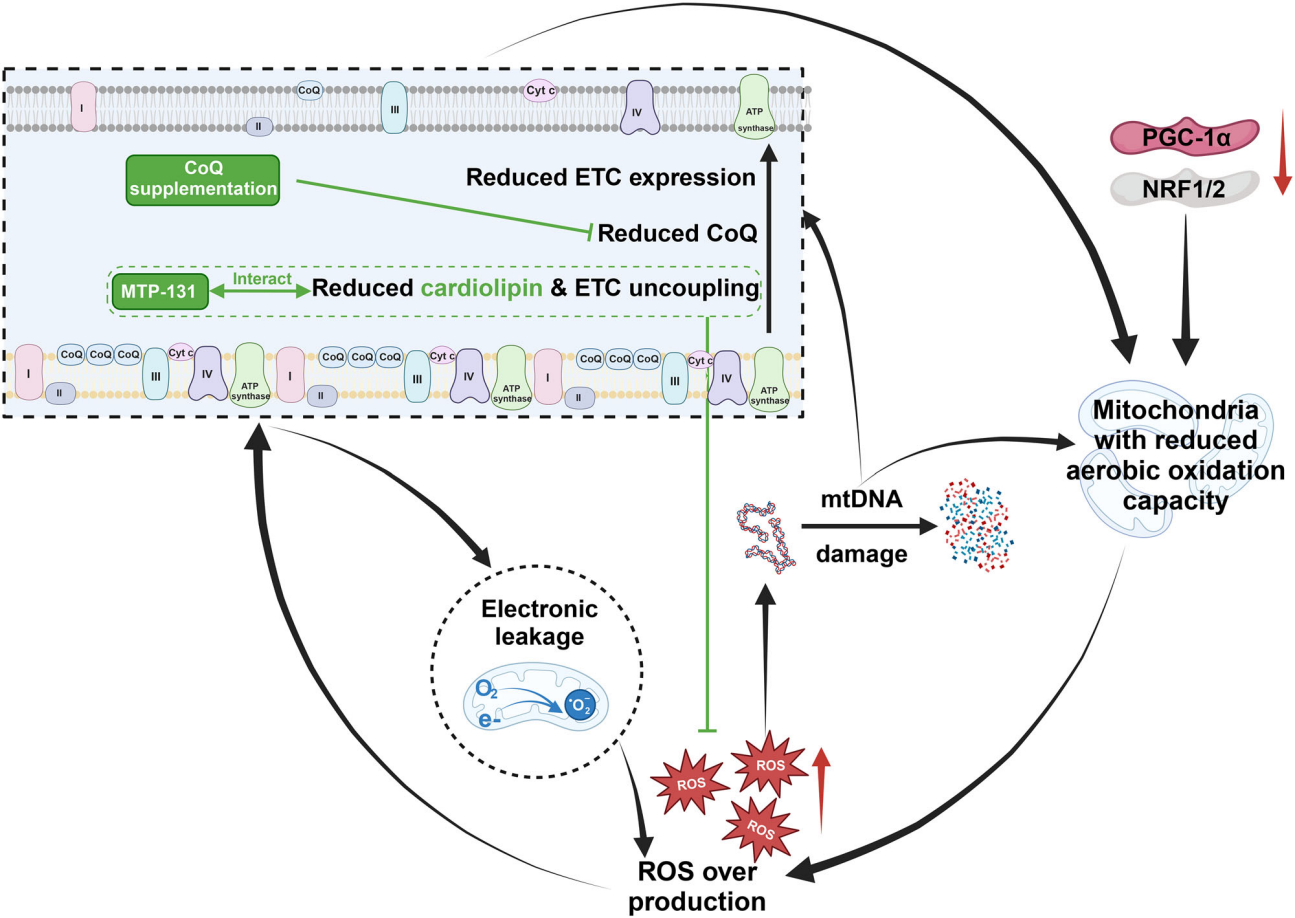
The vicious cycle of mitochondrial damage and ROS production in heart failure. During heart failure, the downregulation of PGC‐1α and NRF1/2 leads to the immaturity of mitochondrial structure and a decline in oxidative capacity, resulting in excessive production of ROS. This excess ROS damages mtDNA, mitochondrial membrane structures, and the ETC, which in turn accelerates ROS production and exacerbates the decline of mitochondrial oxidative function, creating a detrimental cycle. MTP‐131, mitochondrial targeting peptide 131; PGC‐1α, peroxisome proliferator‐activated receptor gamma coactivator 1 alpha; NRF1/2, nuclear respiratory factor 1/2; mtDNA, mitochondrial DNA; ROS, reactive oxygen species; ETC, electron transport chain. Created with BioRender.com, publication license HI288F9E84.

**FIGURE 6:**
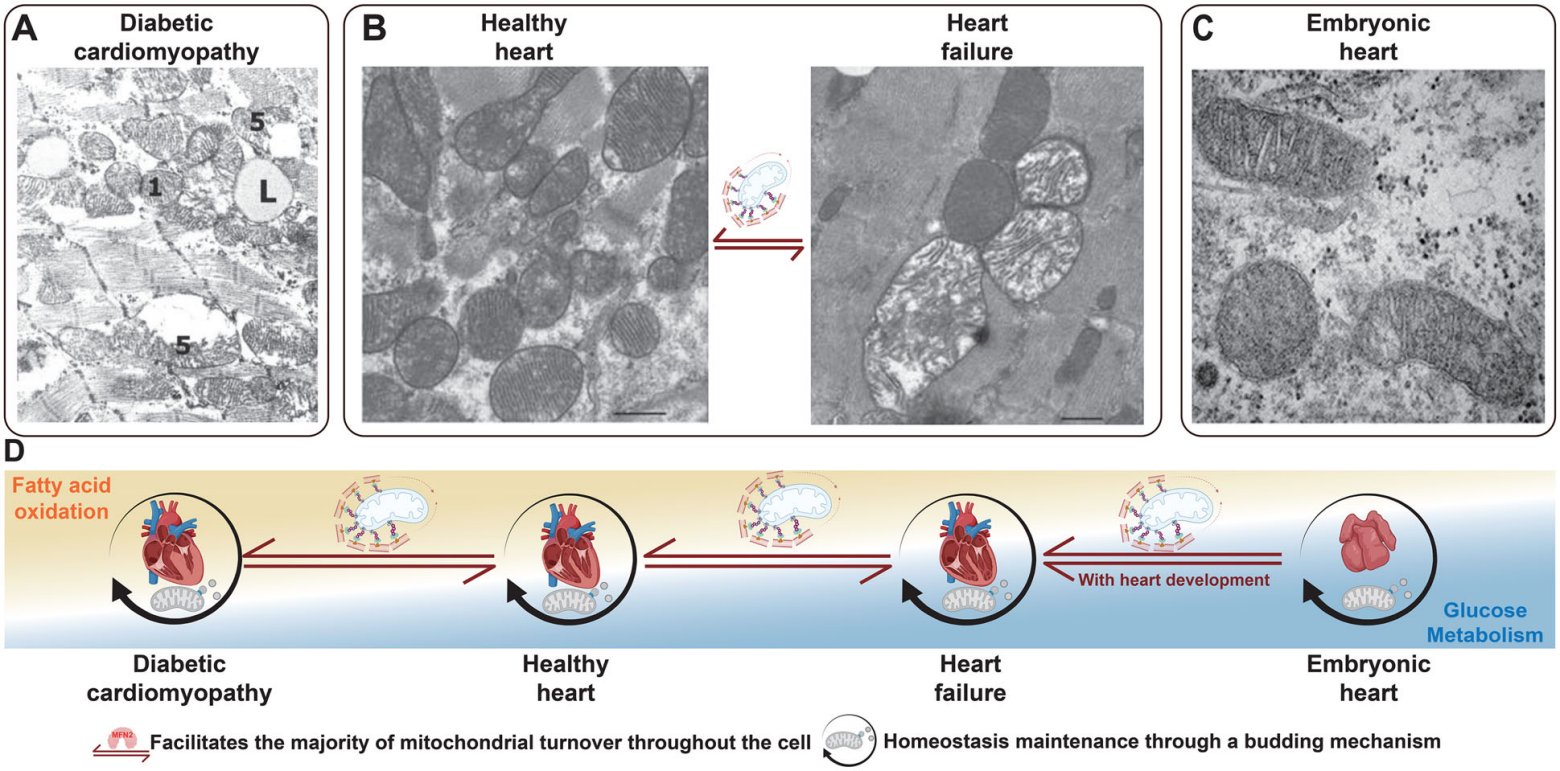
Mitochondria under different metabolic patterns in various physiological and pathological conditions. (A) Transmission electron microscopy (TEM) image of damaged mitochondria accumulated between myofibrils in the cytoplasmic of cardiomyocytes in diabetic cardiomyopathy. (B) TEM images of mitochondria in normal cardiomyocytes and cardiomyocytes in heart failure, showing disorganized cristae and damaged inner membranes in heart failure individuals. (C) Mitochondria in normal embryonic E11.5 cardiomyocytes, characterized by tubular morphology and fewer cristae. (D) From left to right: diabetic cardiomyopathy, healthy heart, heart failure, embryonic heart. These are arranged according to their dependence on glucose metabolism, with the turnover of mitochondria in different metabolic patterns primarily mediated by ubiquitin‐dependent mitophagy. The maintenance of mitochondrial homeostasis within a specific metabolic pattern is mainly reliant on DRP1. A ref. [176], Med Sci Sports Exerc; B ref. [120], Lab Invest; C ref. [58], Development. Created with BioRender.com, publication license VM288FAU9P.

In the context of mitochondrial dysfunction, the primary upstream factor involves the aberrant expression of genes associated with mitochondrial biogenesis and relevant metabolic enzymes. In HF with reduced ejection fraction, pivotal transcription factors such as PGC1α and NRF1/2, which facilitate the positive regulation of genes governing mitochondrial foundational functions, along with a cascade of essential molecules for FAO regulated by PPARα, exhibit significant downregulation [111, 112, 113]. This reduction underpins a systemic decline in the aerobic oxidative metabolic capacity of cardiomyocytes, leading to functional impairments in fatty acid utilization and a metabolic shift from fatty acids to glucose as substrates [114]. As another important regulatory factor in mitochondrial biogenesis during cardiac development, HIF1α plays a dual role in the progression of cardiac diseases, exerting short‐term protection while promoting long‐term disease progression [115]. First, it enhances glycolytic flux by promoting glycolysis‐related enzymes and key genes (e.g., GLUT1, LDHA), aiding myocardial metabolic adaptation and temporarily maintaining ejection fraction and shortening fraction; however, its ability to promote mitochondrial regeneration is quite limited [116]. Furthermore, HIF1α upregulates matrix metalloproteinases while inhibiting tissue inhibitors of metalloproteinases, thereby facilitating ventricular remodeling. It also activates cardiac fibroblasts through the TGF‐β/Smad pathway, promoting collagen deposition and myocardial fibrosis, which contributes to the long‐term progression of the disease and adverse clinical outcomes. In addition to the defects in mitochondrial biogenesis, a range of damaging factors interacts with impaired mitochondria, creating a vicious cycle [117] (Figure 5). Among these factors, the generation of ROS is the most significant contributor. Notably, moderate ROS production does not impair cardiac metabolism and function; rather, it bolsters the ability to counteract oxidative stress and promotes mitochondrial biogenesis. For instance, the underlying mechanism by which moderate exercise improves cardiac function is through the elevation of ROS levels, which in turn enhances the cell's capacity to cope with oxidative stress [118]. However, due to the abnormal mitochondrial biogenesis in cardiomyocytes during HF, defects in the IMM and ETC proteins will lead to electron leakage during the transfer of electrons between different ETC complexes, which results in the generation of a large amount of superoxide anions at the IMM and matrix, exceeding the heart's intrinsic ROS scavenging capacity [119]. Excessive ROS not only damages the antioxidant system, such as NADPH and superoxide dismutase, but also leads to lipid peroxidation, DNA damage, and further injury to the mitochondrial IMM [120] (Figure 6). The reduction of cardiolipin in the mitochondrial inner membrane significantly affects the connection between ETC complexes, severely hindering electron transfer between them [121, 122]. Similarly, the decrease in CoQ also obstructs electron transfer within the ETC, potentially causing electrons to flow backward between ETC complexes. Such damage exacerbates electron leakage and ROS production, creating a vicious cycle (Figure 5). In heart failure, mitochondrial dysfunction initiates a cascade of events, starting with a reduction in the activity of the Trx2 system, which plays a critical role in regulating oxidative stress. This decline in thioredoxin‐2 activity impairs the cell's ability to effectively clear ROS, leading to their accumulation. The excessive ROS further exacerbates mitochondrial damage, disrupting cellular energy production and signaling pathways. This oxidative damage triggers cardiomyocyte apoptosis and hypertrophy, contributing to the progression of heart failure [123]. Progressive imbalance in homeostasis leads to a significant increase in mitochondrial fission, resulting in fragmentation that ultimately activates the mitophagy pathway [124]. While a small amount of mitophagy can help restore metabolic homeostasis under adverse conditions, excessive mitophagy driven by ROS and mitochondrial damage further reduces the number of energy‐producing organelles and disrupts the overall energy supply of the cell [125]. Additionally, abnormalities in mitochondrial biogenesis cause defects in outer mitochondrial membrane proteins that mediate mitophagy, leading to incomplete mitophagy and disruption of the intracellular environment [126]. This incomplete mitophagy can trigger the exacerbation of oxidative stress, activating the heat shock protein heat shock protein family A member 1‐like (HSPA1L), which enhances mitophagy to help maintain cellular homeostasis under adverse conditions [125]. However, excessive mitophagy can lead to apoptotic signaling, prompting the involvement of anti‐apoptotic proteins like Bcl‐2‐associated athanogene proteins 4 (BAG4), which serve to inhibit the recruitment of Parkin and tune the balance between cell survival and apoptosis [127].

Moreover, as the primary metabolic mode of a healthy adult heart (accounting for about 80%), FAO consumes more oxygen to produce a single ATP compared to glycolysis. Therefore, in a hypoxic environment, the relatively inadequate oxygen supply in HF leads to a significant reduction in FAO, while glycolysis increases correspondingly. The metabolic shifts in HF are much less complete compared to the metabolic transformations during the perinatal heart development process. In the fetal stage, FAO and glycolysis each account for 50%. In healthy adult hearts, FAO comprises 80% while glycolysis accounts for 20%. However, in HF patients, FAO still represents 70%, with glycolysis increasing to 30% [56, 128, 129, 130, 131]. Although the 10% upregulation of glycolysis provides some temporary energy supply to the heart, unlike ATP, which is consumed immediately after production, many metabolic intermediates can accumulate in the cytoplasm. The accumulation of glycolytic intermediates and substrates can further exacerbate cardiac remodeling and impair the oxidative metabolism of cardiomyocytes through interference with related proteins and pathways [132]. This suggests that the metabolic shift acts more like “drinking poison to quench thirst,” representing a maladaptation rather than a true adaptation (Figure 4).

Among the metabolic switches, alterations in glucose metabolism occur first. This is primarily characterized by an increase in glucose uptake and glycolysis, alongside a reduction in aerobic oxidation of glucose [133] (Figure 4). Consistent with the reduction in FAO, significant inhibition of aerobic glucose oxidation has been observed in both patients and various animal models due to mitochondrial dysfunction. In HF, there is a significant downregulation of mitochondrial pyruvate dehydrogenase (PDH) and mitochondrial pyruvate carrier (MPC) [134]. Additionally, the upregulation of pyruvate dehydrogenase kinase 4 (PDK4) inhibits PDH through phosphorylation [135]. Furthermore, the fetal isoform of pyruvate kinase 2 is markedly increased compared to the adult isoform pyruvate kinase 1, leading to the suppression of the conversion of phosphoenolpyruvate to pyruvate [136]. These changes indicate that the generation of pyruvate and its entry into the mitochondria are significantly reduced in cardiomyocytes upon HF. Although a small fraction of pyruvate manages to enter the mitochondria, this does not necessarily imply its fate is to enter the TCA cycle. In cardiomyocytes affected by HF, there is a marked upregulation of anaplerotic enzymes, such as malic enzyme 1 (ME1). Consequently, a portion of the pyruvate is redirected toward the synthesis of TCA cycle intermediates [137]. The generated oxaloacetate can subsequently be converted into aspartate, providing critical substrates that contribute to cardiac hypertrophy in these cells [24]. The upregulation of anaplerosis not only leads to a reduction in glucose availability for ATP production but also results in the depletion of NADPH, which exacerbates oxidative stress and cellular damage [138]. This metabolic reprogramming underscores the altered energetic landscape in HF, where pathways that support growth and remodeling are favored over traditional oxidative phosphorylation.

Due to increased glucose uptake but reduced aerobic oxidation, a large amount of glucose exists in the cells in the form of itself and glycolytic intermediates along with their bypass products, leading to myocardial hypertrophy and remodeling. Intracellular glucose inhibits CREB‐regulated KLF transcription, which not only suppresses mitochondrial biogenesis but also activates mTOR, forming an important signal for cardiac hypertrophy [139]. Additionally, glucose‐6‐phosphate (G6P) can directly activate mTOR, further promoting myocardial hypertrophy [140]. As a starting point for multiple glycolytic branching metabolic pathways, G6P can enter the PPP and polyol pathway, thereby activating MAPK and other hypertrophy‐related pathways, while also providing raw materials for the resynthesis of components required for hypertrophy [141]. The hexosamine pathway, derived from fructose‐6‐phosphate (F6P), primarily regulates gene transcription and pathway activation through O‐GlcNAc modification [142]. This pathway can upregulate MAPK pathway activity and enhance the activity of certain transcription factors, such as GATA4, thereby promoting the expression of genes related to cell proliferation and hypertrophy, such as β‐myosin heavy chain and B‐type natriuretic peptide [143]. As downstream products of anaerobic glycolysis, the large amounts of hydrogen ions released by pyruvate and lactate can lead to a surge in intracellular calcium levels through sodium‐hydrogen exchange and sodium‐calcium exchange, potentially activating cell death‐related pathways [144, 145] (Figure 4).

In addition to influencing cardiac remodeling in various aspects and exacerbating cardiomyocyte hypertrophy and impairments in oxidative metabolism, glycolysis and the resultant redundancy of its metabolic intermediates are increasingly recognized for their critical role in modulating the susceptibility of cardiomyocytes to oxidative stress‐induced damage. The adoption of a ketogenic diet leads to the suppression of glycolysis and its non‐oxidative metabolic pathways. Such modulation can effectively slow or even prevent the onset of HF when induced via transverse aortic constriction. Conversely, initiating a ketogenic diet after the onset of HF fails to yield any therapeutic benefits [146]. This underscores the pivotal role of glycolysis and its bypass metabolic intermediates in the pathogenesis and progression of HF.

Under the influence of glucose metabolism in driving myocardial remodeling, the myocardium progresses from a state of compensatory hypertrophy to one of decompensation, characterized by a gradual decline in ejection fraction. As the ejection fraction progressively declines to 50%, there is a pronounced reduction in FAO, which frequently corresponds with disruptions in the transcriptional regulatory functions of PGC1α and PPARα [147, 148]. As the heart is one of the primary organs for fatty acid consumption in the body, an increase in serum fatty acids often indicates metabolic abnormalities and energy deficits within the myocardium. This elevation may serve as a biomarker for myocardial dysfunction and, when considered alongside other risk factors, can provide valuable insights into the potential onset of HF [149, 150]. Elevated serum fatty acids can act as a form of “excess fuel,” stimulating FAO in the heart. However, in cells where mitochondrial abundance and oxidative metabolic capacity are significantly diminished, this stimulation does not improve the metabolic state. Instead, it exacerbates oxidative stress within the cells, further contributing to myocardial dysfunction and complicating the pathophysiology of HF [151]. At this stage, implementing a series of strategies to inhibit FAO, such as using β‐blockers or inhibiting the decarboxylation of malonyl‐CoA, can help alleviate oxidative stress in the cells [152, 153]. These approaches promote aerobic glycolysis, thereby not only preventing the accelerated disruption of myocardial cell homeostasis but also reducing the accumulation of glycolytic and intermediate metabolites associated with maladaptive remodeling. This shift can facilitate a transition from maladaptation to a more adaptive metabolic state within the myocardium [154] (Figure 4).

In conclusion, it is inappropriate to simply regard the shift in glucose metabolism and the impairment of FAO as the cause and effect of HF [155]. It is more likely that the two metabolic pathways play distinct roles at different stages of HF progression. Intervening in the corresponding metabolism at specific stages may offer a more personalized approach to HF treatment. Additionally, enhancing mitochondria, the core organelles of energy metabolism, appears to be a more universal strategy for treating HF, which warrants further research [156].

### 3.2 Diabetic Cardiomyopathy

As a significant risk factor for primary cardiovascular diseases, diabetes itself can precipitate DCM, which is underpinned by cardiac metabolic dysfunction [157, 158]. The pathological manifestations predominantly include myocardial hypertrophy, myocardial fibrosis, and diastolic dysfunction, resembling the alterations observed in hypertrophic and restrictive cardiomyopathies [159]. In contrast to the “starvation” state experienced by cardiomyocytes in HF, those in the context of DCM exist within an extracellular environment marked by elevated glucose and free fatty acids [160]. In insulin‐resistant cardiomyocytes, this excess of substrates fails to improve cardiac metabolism; instead, it precipitates profound metabolic dysregulation and significant impairments in energy supply (Figure 4).

#### 3.2.1 Imbalance in Mitochondrial Dynamics

Under the stimulation of elevated glucose and free fatty acids, cardiomyocytes in patients with Type 2 Diabetes Mellitus (T2DM) exhibit an early compensatory response characterized by the upregulation of transcription factors such as PGC1α, leading to enhanced mitochondrial biogenesis [161]. This adaptive change helps cardiomyocytes cope with metabolic stress. Moreover, multiple factors play a critical role in maintaining mitochondrial homeostasis, and the dynamics of mitochondrial proteins such as DRP1, OPA1, and MFN2 can become aberrant under the influence of various signaling pathways and post‐translational modifications [162, 163]. This disruption leads to an imbalance between mitochondrial fission and fusion processes. Additionally, mitochondrial autophagy, which is mediated by outer mitochondrial membrane proteins like MFN2, is profoundly impaired as a result. Excessive mitochondrial fission leads to a reduction in the area of IMM, compromising the organelles' capacity to manage excess metabolic substrates and resulting in an overproduction of ROS. This ROS overload, akin to that observed in HF, inflicts damage on the IMM. Additionally, in DCM, the aberrant proteins on the OMM lead to the impairment of mitophagy mechanisms, hindering the effective clearance of dysfunctional mitochondria, which would typically be eliminated through ubiquitously induced mitophagy [164] (Figure 6). The presence of damaged mitochondrial membranes leads to a significant leakage of mtDNA into the cytosolic matrix, where it is recognized by cyclic GMP‐AMP synthase, subsequently activating the stimulator of interferon genes pathway [165, 166]. This interaction triggers the activation of downstream transcription factors such as NF‐κB and interferon regulatory factor 3, promoting inflammatory responses and apoptosis [167]. This cascade exacerbates cellular stress, further compromising cardiac function [168] (Figure 4).

#### 3.2.2 Disruption of Calcium Homeostasis

Calcium homeostasis disruption is also a significant contributor to the pathogenesis and progression of DCM, with a critical factor being the reduced levels of the mitochondrial calcium uniporter (MCU) [169]. The MCU, functioning as a transporter protein on the IMM, plays a crucial role in the uptake of calcium ions essential for mitochondrial function. A significant reduction in MCU levels disrupts this process, resulting in diminished calcium concentrations within the mitochondria and an elevation of calcium levels in the cytosol [170]. The former results in the reduced activity of various metabolic enzymes, impacting energy production and serving as a molecular basis for the substrate preference shift during the pathogenesis of DCM. The latter alters calcium signaling within cardiomyocytes, promoting apoptotic pathways that ultimately impair cardiac contractility, leading to contractile dysfunction in the late stages of DCM. Calcium transport mediated by the inositol trisphosphate receptor and the VDAC plays a pivotal role in maintaining calcium homeostasis between the endoplasmic reticulum and mitochondria [171, 172]. In the context of DCM, the downregulation of this pathway further exacerbates mitochondrial calcium reduction. Moreover, the abnormal activation of ORAI calcium release‐activated calcium channel 1 in DCM leads to an increased influx of calcium ions, which subsequently raises intracellular calcium concentrations [173]. This elevation activates calcium‐dependent signaling pathways, such as extracellular signal‐regulated kinase 1, further modulating the phosphorylation status of DRP1 and promoting its role in mediating mitochondrial fission. Beyond enhancing mitochondrial fission, the upregulation of DRP1 also activates mammalian STE20‐like kinase 1, which subsequently exerts inhibitory effects on Parkin levels via Sirtuin 3. This reduction in Parkin activity hampers mitophagy, leading to the accumulation of dysfunctional mitochondria within the cytoplasm [174, 175, 176]. Overexpression of Sirtuin 3 in vitro can enhance the recruitment of Parkin and facilitate mitophagy by upregulating the expression of key adaptor proteins such as PINK, Parkin, and p62. This process ultimately helps to prevent further disruptions in the homeostasis of cardiomyocytes [177]. The administration of Tat‐Beclin1 facilitates the activation of intracellular Beclin1 through its interaction with GTPase‐activating protein related 1, a negative regulator of mitophagy [178]. This interaction effectively induces autophagy, thereby enhancing the degradation of damaged or dysfunctional mitochondria. Moreover, the upregulation of miR‐30c in db/db mice inhibits anti‐apoptotic proteins like Bcl‐2, which subsequently enhances mitophagy and leads to improvements in myocardial cell metabolism and function [179]. The aforementioned studies demonstrating the enhancement of mitophagy to improve DCM further underscore the pivotal role of impaired mitophagy in the pathogenesis and progression of DCM.

#### 3.2.3 Alterations in Substrate Preferences

DCM is characterized by altered substrate preferences in energy metabolism, notably a reduced reliance on glucose metabolism and an increased dependence on FAO [3]. This metabolic shift manifests as downregulation across the entire glucose metabolic pathway, including uptake, glycolysis, and oxidation. A key causation associated with such metabolic shift is insulin resistance (IR), a crucial mechanism of diabetes.

In the early stages of IR, pathways such as mTOR/ S6 kinase 1 may partially compensate for the impaired insulin signaling, promoting glucose utilization and improving cardiac function. However, as DCM progresses, the progressive decline of insulin signaling significantly impacts substrate uptake [180].

In a healthy heart, activation of the insulin signaling pathway insulin receptor substrate‐phosphoinositide 3 kinase‐protein kinase B, leads to the translocation of glucose transporter 4 (GLUT4) from the cytoplasm to the cell membrane, while fatty acid transporter cluster of differentiation 36 (CD36) is internalized [181, 182]. This translocation is critical for facilitating insulin‐mediated glucose uptake. In DCM, IR markedly decreases the activity of the insulin signaling pathway, resulting in reduced GLUT4 and increased CD36 levels at the cell membrane. This shift significantly enhances fatty acid uptake while inhibiting glucose uptake, creating a foundation for the abnormal decrease in glucose metabolism and the elevation of FAO (Figure 4). Studies have shown that bariatric surgery in rats can partially reverse IR and its associated GLUT4 mislocalization, thereby improving glucose metabolism levels in DCM cardiomyocytes [183]. This further emphasizes the crucial role of transporter mislocalization in the progression of DCM.

Abnormal insulin signaling leads to a reduction in the activity of the key glycolytic enzyme, phosphofructokinase. Additionally, excessive fatty acid uptake mediated by CD36 promotes the phosphorylation and reduced activity of the key enzyme PDH through the upregulation of PDK. These enzymatic changes ultimately result in the accumulation of glucose in the cytoplasm in the form of G6P, F6P, and its own form [184, 185].

Excess glucose can undergo glycation to form advanced glycation end products (AGEs), which subsequently bind to their receptor RAGE [186]. This binding initiates a cascade of signaling pathways, including NF‐κB and MAPK pathways, ultimately driving the expression of numerous inflammatory cytokines and collagen [187]. This process contributes to the development of chronic inflammation and tissue fibrosis. AGEs can form cross‐links with collagen, which enhances its stability and resistance to degradation, thereby perpetuating a fibrotic phenotype [188]. Concurrently, the glycolytic intermediate G6P is capable of activating inflammatory pathways, including NF‐κB. Moreover, AGEs can enhance the flow of the hexosamine pathway, resulting in O‐GlcNAc modification of key proteins, including protein kinase B (AKT), DRP1, and MFN2 [189]. This modification not only aggravates insulin signaling impairment and mitochondrial structural abnormality but also diminishes the anti‐apoptotic function of AKT, thereby exacerbating myocardial injury [190]. Furthermore, G6P can be channeled through the pentose phosphate pathway, supplying crucial substrates that facilitate the processes associated with cardiac hypertrophy. In summary, impaired glucose metabolism leads to the accumulation of various metabolic intermediates within the cells, promoting myocardial inflammation and cardiomyocyte apoptosis [191]. This process subsequently results in the development of myocardial hypertrophy and fibrosis in patients with diabetes mellitus (Figure 4).

During the onset of diabetes, organs such as the liver and adipose tissue, which are responsible for lipid uptake for metabolism and storage, also become affected and exhibit IR. This leads to an increase in circulating fatty acids in patients. This increase, along with excessive fatty acid intake mediated by CD36, will lead to enhanced FAO, resulting in a greater proportion of FAO in cardiac metabolism [192]. Such a metabolic shift appears to provide an alternative substrate in the context of glucose utilization impairment caused by IR. However, the reliance on less efficient FAO instead of the original glucose metabolism can lead to increased production of ROS, further exacerbating existing mitochondrial damage and intensifying oxidative stress in cardiomyocytes [193].

In summary, during the onset of diabetes, mitochondrial dysfunction occurs in cardiomyocytes. IR leads to a shift in substrate preference in the myocardium through the translocation of transporters. This metabolic shift is considered a maladaptation, where impaired glucose metabolism and upregulation of FAO synergistically exacerbate IR, resulting in energy supply deficits in cardiomyocytes, as well as cardiac hypertrophy and fibrosis.

## 4 Conclusion

In conclusion, prior research indicates that during cardiac development, mitochondria function as sensors for fluctuations in cellular energy status and regulators of metabolic transitions. They detect the supply of oxygen and nutrients, responding through mechanisms such as biogenesis, fission and fusion, and mitophagy, thereby facilitating metabolic changes during the perinatal period. During metabolic transitions, a variety of metabolic intermediates and ROS are generated. These molecules not only fail to adversely affect cellular function but can also be harnessed by cells to regulate alterations in gene expression patterns through epigenetic mechanisms. Moreover, mild and controlled oxidative stress can facilitate the maturation of neonatal cardiomyocytes.

In the context of various cardiovascular diseases, mitochondria also respond to fluctuations in fuel supply. However, this response stands in contrast to the “advantageous and harmless” effects observed during the perinatal period, where mitochondrial adaptations are essential for sustaining cardiomyocyte survival during transient energy deprivation and facilitating their further maturation. Although the changes in mitochondria under pathological conditions that mediate shifts in metabolic substrate preferences are crucial for cellular function, they do play a significant role in temporarily improving energy supply to cardiomyocytes. Nonetheless, the array of metabolic products, intermediates, and substrates resulting from these metabolic shifts can modulate the activity of pertinent signaling pathways and transcription factors, thereby exerting a significant influence on cardiomyocyte remodeling and the progression of cardiovascular diseases. As the metabolic role of mitochondria in the pathogenesis and progression of cardiac diseases is being elucidated, the mitochondrial interventions of traditional cardiovascular drugs and therapies are gradually being recognized. For example, in addition to their traditional mechanism of reducing sympathetic tone, recent studies suggest that beta‐blockers may also protect mitochondria by inhibiting MCU‐mediated excessive calcium influx, modulating the opening degree of the mitochondrial permeability transition pore, and preventing mitochondrial calcium overload [194, 195]. In addition, drugs targeting mitochondria are increasingly being developed (Supporting information S1).

While significant progress has been made in understanding how mitochondria regulate cardiomyocyte development and disease‐related alterations, key questions remain. The extent to which intrinsic changes in mitochondria influence cellular gene expression patterns is still unclear. Additionally, whether adverse environmental exposure during the perinatal period may affect normal metabolic transitions, potentially modulating susceptibility to cardiovascular diseases in adulthood. These critical issues require further investigation.

## Conflicts of Interest

The authors declare no conflicts of interest.

## Supporting information




**Supporting File**: exp270157‐sup‐0001‐SuppMat.pdf.

## Data Availability

The data that support the findings of this study are available from the corresponding author upon reasonable request.
