# Supplementary material for: The Metabolic Role of Mitochondria in the Perinatal Cardiac Development and Cardiovascular Diseases
Source: Exploration (Beijing). 2026 Mar 31;6(2):20240414. doi: 10.1002/EXP.20240414 (PMC13094534; doi:10.1002/EXP.20240414)
Supplement: Supplementary file 1 — Supporting File: exp270157‐sup‐0001‐SuppMat.pdf. [file EXP2-6-20240414-s001.pdf]

Supplementary information 1.

To address numerous cardiovascular diseases resulting from mitochondrial morphological and functional dysregulation, various mitochondria-targeting therapeutics have been developed. These agents modulate distinct signaling pathways to ameliorate mitochondrial fragmentation and reduction in mitochondrial quantity (Table 1).

Table1. Mechanisms and Recent Advances in Mitochondria-Targeting Cardiovascular drugs

Drugs	Mechanisms	Research Stage	Reference
Mdivi-1	Inhibiting Drp1-mediated excessive mitochondrial fission to maintain mitochondrial network integrity	Preclinical stage (animal/cell-based studies)	[1]
Urolithin A	Activating the PINK1/Parkin pathway to clear damaged mitochondria and promote new mitochondrial generation	Clinical trials have confirmed its improvement in cardiovascular risk markers	[2,3]
Elamipretid (SS-31 peptide)	Bind to cardiolipin to stabilize the mitochondrial inner membrane, reduce the generation of ROS, and improve energy metabolism	Phase II clinical trial targeting chronic heart failure	[4,5]
epicatechin	Activating PGC-1α and TFAM to increase mitochondrial number; directly scavenging reactive oxygen species (ROS); reducing Drp1 phosphorylation to decrease mitochondrial fragmentation.	Clinical trials related to prevention of cardiovascular disease	[6]
Resveratrol	Activate the SIRT1/PGC-1α pathway to promote mitochondrial biogenesis	Phase II clinical trial	[7,8]

Additionally, as discussed in the main text, the metabolic profiles of cardiomyocytes and other cardiac-resident cells undergo significant alterations under pathological conditions[9]. For instance, cardiomyocytes shift toward glucose-dependent metabolism in heart failure, while relying predominantly on lipids in diabetic cardiomyopathy[10]. These pathological metabolic patterns substantially influence the epigenetic landscape of proliferation- and contractility-related genes in cardiomyocytes through metabolite-driven mechanisms, ultimately contributing to disease

pathogenesis[11]. In therapeutic development, a promising avenue lies in implementing preemptive mitochondrial-targeting interventions during early-stage metabolic dysregulation. By specifically targeting epigenetic-modulating metabolites, this strategy could prevent the emergence of pathological gene expression patterns.

Reference:

[1] C. Piamsiri, C. Maneechote, K. Jinawong, B. Arunsak, T. Chunchai, W. Nawara, S. Kerdphoo, S. C. Chattipakorn, N. Chattipakorn, *Eur J Pharmacol* **2024**,977,176736.

[2] S. Liu, J. Faitg, C. Tissot, D. Konstantopoulos, R. Laws, G. Bourdier, P. A. Andreux, T. Davey, H. Gallart-Ayala, J. Ivanisevic, A. Singh, C. Rinsch, D. J. Marcinek, D. D'Amico, *iScience* **2025**,28,111814.

[3] S. Liu, D. D'Amico, E. Shankland, S. Bhayana, J. M. Garcia, P. Aebischer, C. Rinsch, A. Singh, D. J. Marcinek, *JAMA Netw Open* **2022**,5,e2144279.

[4] J. Butler, M. S. Khan, S. D. Anker, G. C. Fonarow, R. J. Kim, S. Nodari, C. M. O'Connor, B. Pieske, E. Pieske-Kraigher, H. N. Sabbah, M. Senni, A. A. Voors, J. E. Udelson, J. Carr, M. Gheorghiade, G. Filippatos, *J Card Fail* **2020**,26,429.

[5] M. A. Daubert, E. Yow, G. Dunn, S. Marchev, H. Barnhart, P. S. Douglas, C. O'Connor, S. Goldstein, J. E. Udelson, H. N. Sabbah, *Circ Heart Fail* **2017**,10,

[6] H. D. Sesso, J. E. Manson, A. K. Aragaki, P. M. Rist, L. G. Johnson, G. Friedenberg, T. Copeland, A. Clar, S. Mora, M. V. Moorthy, A. Sarkissian, W. R. Carrick, G. L. Anderson, *Am J Clin Nutr* **2022**,115,1490.

[7] R. Gal, D. Praksch, P. Kenyeres, M. Rabai, K. Toth, R. Halmosi, T. Habon, *Cardiovasc Ther* **2020**,2020,7262474.

[8] J. H. Storgaard, N. Løkken, K. L. Madsen, N. C. Voermans, P. Laforêt, A. Nadaj-Pakleza, C. Tard, G. van Hall, J. Vissing, M. C. Ørngreen, *J Inherit Metab Dis* **2022**,45,517.

[9] M. Alexanian, A. Padmanabhan, T. Nishino, J. G. Travers, L. Ye, A. Pelonero, C. Y. Lee, N. Sadagopan, Y. Huang, K. Auclair, A. Zhu, Y. An, C. A. Ekstrand, C. Martinez, B. G. Teran, W. R. Flanigan, C. K. Kim, K. Lumbao-Conradson, Z. Gardner, L. Li, M. W. Costa, R. Jain, I. Charo, A. J. Combes, S. M. Haldar, K. S. Pollard, R. J. Vagnozzi, T. A. McKinsey, P. F. Przytycki, D. Srivastava, *Nature* **2024**,635,434.

[10] Q. Sun, Q. G. Karwi, N. Wong, G. D. Lopaschuk, *Cardiovasc Res* **2024**,120,1996.

[11] M. Nakamura, J. Sadoshima, *Nat Rev Cardiol* **2018**,15,387.
